# Integrated analysis of bacterial and microeukaryotic communities from differentially active mud volcanoes in the Gulf of Cadiz

**DOI:** 10.1038/srep35272

**Published:** 2016-10-20

**Authors:** Francisco J. R. C. Coelho, António Louvado, Patrícia M. Domingues, Daniel F. R. Cleary, Marina Ferreira, Adelaide Almeida, Marina R. Cunha, Ângela Cunha, Newton C. M. Gomes

**Affiliations:** 1 Department of Biology & CESAM, University of Aveiro, Campus de Santiago, 3810-193 Aveiro, Portugal; 2 Department of Chemistry & CICECO, University of Aveiro, Campus de Santiago, 3810-193 Aveiro, Portugal

## Abstract

The present study assesses the diversity and composition of sediment bacterial and microeukaryotic communities from deep-sea mud volcanoes (MVs) associated with strike-slip faults in the South-West Iberian Margin (SWIM). We used a 16S/18S rRNA gene based pyrosequencing approach to characterize and correlate the sediment bacterial and microeukaryotic communities from MVs with differing gas seep regimes and from an additional site with no apparent seeping activity. In general, our results showed significant compositional changes of bacterial and microeukaryotic communities in sampling sites with different seepage regimes. Sediment bacterial communities were enriched with *Methylococcales* (putative methanotrophs) but had lower abundances of *Rhodospirillales*, *Nitrospirales* and SAR202 in the more active MVs. Within microeukaryotic communities, members of the *Lobosa* (lobose amoebae) were enriched in more active MVs. We also showed a strong correlation between *Methylococcales* populations and lobose amoeba in active MVs. This study provides baseline information on the diversity and composition of bacterial and microeukaryotic communities in deep-sea MVs associated with strike-slip faults.

Interactions between the Eurasian and African plates at the SWIM have resulted in a complex geological setting characterized by the extensive occurrence of mud volcanoes (MVs), at depths between 200 m and 5000 m^1^,^2^,^3^. MVs are piercement structures, normally related to tectonic activity or petroleum reservoirs and are usually created by compression forces that promote the intrusion and seepage of pressurized fluids composed of gas, water, liquid hydrocarbons and solid particles^4^. Methane is the most predominant gas expelled at MVs, which together with methane released from other types of seeps, increases atmospheric carbon content by 0.01–0.05 Gt yr^−1^, accounting for 1 to 5% of global methane emissions to the atmosphere^5^.

Methane-oxidizing microorganisms mediate the methane flux in these ecosystems; providing energy sources to other microorganisms through predation and symbiosis, thus generating a hotspot of biomass in the deep-sea^6^. Together, micro-and macroorganisms at methane seeps consume *c.* 75% of the methane that reaches the seafloor; thus serving as a biological filter that controls the emission of this greenhouse gas from the ocean to the atmosphere^5^,^7^. In surface sediments of MV seeps, methane oxidation is usually performed by aerobic methanotrophic bacteria^8^. In the subsurface deeper sediments it is mainly accomplished by anaerobic methanotrophic archaea in association with sulfate reducing bacteria, adding another reduced compound (hydrogen sulfide) to the seeping fluids^9^,^10^. Some methanotrophic archaea and thiotrophic bacteria form symbiotic relationships with macroeukaryotes, such as tubeworms and clams^9^,^11^.

There is evidence that the methane released by several MVs piercing a thick accretionary wedge in the Gulf of Cadiz (AWGC, SWIM) is almost totally consumed at the seabed, and therefore does not constitute a relevant source of methane to the hydrosphere and subsequently to the atmosphere^10^. This is a strong indication of the presence of developed benthic communities involved in the uptake of methane. Although several studies have surveyed the microbial communities within MVs^10^,^12^,^13^, there is still considerable uncertainty regarding their structural and functional composition, in particular in regard to microeukaryotes - the first-level consumers. In this study, a 16S and 18S-based pyrosequencing approach was applied to investigate prokaryotic and microeukaryotic communities present in the surface sediments of deep-sea MVs associated with strike-slip faults in the SWIM. Within the prokaryotic communities, we focused our analysis on *Bacteria*, assuming their high abundance and functional relevance in the process of methane oxidation in surface sediments of MV seeps^14^,^15^. Our main objectives were to characterize the bacterial and microeukaryotic communities in MVs with different seepage regimes and to assess possible relationships between different microbial groups. Ultimately, this work can contribute to the understanding of microbial communities and their potential impact on processes in the MV environment.

## Material and Methods

### Study sites and sampling

**Figure 1:**
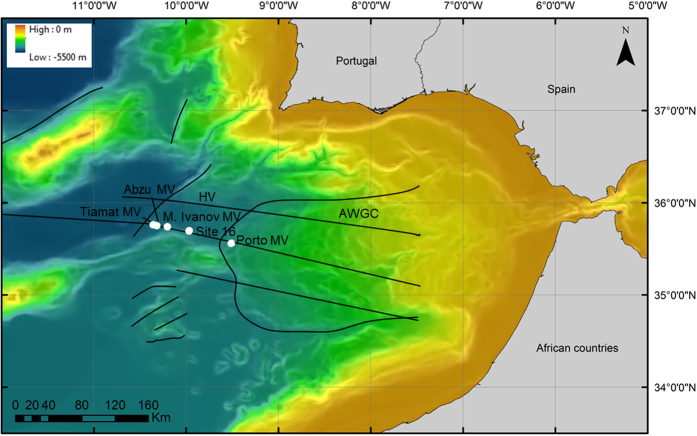
Overview of the studied sites (Tiamat - 339; Abzu - 349, 369; SE M.Ivanov - 329, 388 and 407, NW M. Ivanov - 348, site 16-307 and Porto-308). The maps were generated using ArcGIS 10.0 (version 10.0; http://www.esri.com/) software with bathymetry data previously published^16^.

The SWIM is a region with a complex geological history and important tectonic activity related to the Africa-Eurasia plate boundary. The tectonic deformation is accommodated by the right-lateral SWIM strike-slip faults^16^ and by the AWGC underlain by a strip of old (140 My) oceanic crust^17^. The SWIM faults cut through most of the lithosphere and are the main pathways for fluid percolation, leading to the development of seepage areas frequently expressed as some of the most active MVs in the Gulf of Cadiz^18^,^19^. Mud volcanism in the SWIM was first reported at the AWGC, where over 40 MVs have been located and sampled at depths between 200 and 4000 m^1^,^20^. During the SWIMGLO/Transflux M86/5 cruise onboard the RV Meteor (leg 5, 23 February to 16 March 2012), three new mud volcanoes (Abzu, Tiamat and Mikhail Ivanov) were discovered sitting on the SWIM1 fault at the Horseshoe Valley, c. ~90 km west of the deformation front of the AWGC (Fig. 1)^3^,^21^. These MVs are intimately connected to the SWIM faults and are characterized by a chemical signature indicative of fluid sources from oceanic crust older than 140 Ma^3^.

In this study, samples were taken from Abzu, Tiamat, Mikhail Ivanov and from the Porto MV, both located on the AWGC. A greater sampling effort was allocated to M. Ivanov MV which is formed by a complex of several craters: the northwest (NW) crater is, at present, apparently inactive while the gas hydrates collected from the southeast (SE) crater indicate higher seepage activity than in the other sampled MVs. This combination of sampling sites allowed us to distinguish among three different degrees (relative scale) of seepage activity based on the observed differences in the thickness of the overlaying hemipelagic layer^21^ and the presence or absence of chemotrophic fauna (well-known indicators of seepage activity^1^,^6^,^20^,^22^) in the collected samples (M.R. Cunha, personal observation): (i) M. Ivanov MV, SE crater (stations 329, 388, 407) – the most active, characterized by the presence of mud breccia, gas hydrates and the presence of chemotrophic fauna such as frenulate worms, and solemyid and vesicomyid bivalves confirmed by boxcore sampling and/or AUV photo surveys; (ii) Abzu and Tiamat (stations 339, 349, 369) – activity confirmed by the presence of chemotrophic fauna (frenulates and solemyids) and mud breccia (although covered by hemipelagic sediments); and (iii) M. Ivanov MV, NW crater –(station 348) – apparently inactive, boxcore sampling recovered mostly hemipelagic sediments and no chemotrophic fauna. Porto MV (station 308) has already been confirmed as an active MV during previous investigations^22^. Furthermore, site 16 (station 307, not a MV), an additional inactive site, was sampled for reference. The sample collection included a total of seven sediment samples taken with a boxcorer and two with a multi corer (Fig. 1 and Table 1). Sub-samples of 0.5–1 g of superficial sediments (0–1 cm bsf) were collected and deep-frozen (−80 °C) immediately following collection, kept on dry ice during transportation, and stored at −80 °C until further analysis.

### DNA extraction and bacterial and microeukaryotic community analysis

For DNA extraction, four sections of sediment collected from the same core (0.25 g each) were combined into 2.0 ml screw cap Lysing Matrix tubes of the FastDNA^®^ Spin Kit for Soil (MP Biomedicals, CA, USA). The DNA was extracted according to the manufacturer’s guidelines. The extraction protocol included an initial homogenization using a FastPrep^®^ Instrument (MP Biomedicals, CA, USA), for 40 s at a speed setting of 6.0 ms^−1^.

A 16S and 18S rRNA based barcoded pyrosequencing approach was used to characterize both the bacterial and microeukaryotic communities from each site. For bacterial community analysis, *c.* 525 bp fragments of the 16S rRNA were sequenced for each sample with primers V3 Forward (5′-ACTCCTACGGGAGGCAG-3′) and V4 Reverse (5′-TACNVRRGTHTCTAATYC-3′) with Roche 454 titanium sequencing adapters^23^. The 454 pyrosequencing profiling of microeukaryotic communities was achieved via amplification of *c.* 450 bp 18S small subunit rRNA gene fragments using primers SSUFO4 (5′-GCTTGTCTCAAAGATTAAGCC-3′) and SSU_R22 (5′-GCCTGCTGCCTTCCTTGGA-3′) and cycling conditions as previously described^24^. Equimolar concentrations of the PCR products were then sequenced using GS 454 FLX Titanium chemistry according to manufacturers instructions (Roche, 454 Life Sciences, Brandford, CT, USA). Sequences can be downloaded from the NCBI Short Read Archive (study accessions PRJNA305416).

Both barcoded pyrosequencing libraries (bacterial and microeukaryotic) were processed using the Quantitative Insights Into Microbial Ecology (QIIME) software package (http://qiime.org; Accessed 01 January 2014) according to published recommendations and following previously described methods^25^,^26^ with the exception of the OTU picking step, where the UPARSE^27^ clustering method and chimera check was used. In QIIME, fasta and qual files were used as input for the split_libraries.py script. Default arguments were used except for the minimum sequence length, which was set at 218 bps after removal of forward primers and barcodes; reverse primers were removed using the ‘truncate only’ argument and a sliding window test of quality scores was enabled with a value of 50 as suggested in the QIIME description for the script. OTUs were selected using UPARSE with usearch7^27^. Chimera checking was performed using the UCHIME algorithm. First reads were filtered with the -fastq_filter command and the following arguments -fastq_trunclen 250, -fastq_maxee 0.5 and -fastq_truncqual 15. Sequences were then dereplicated and sorted using the -derep_fulllength and - sortbysize commands. OTU clustering was performed using the -cluster_otus command (cut-off threshold at 97%). An additional chimera check was subsequently applied using the -uchime_ref command with the gold.fa database (http://drive5.com/uchime/gold.fa). In QIIME, representative sequences were selected using the pick_rep_set.py script in QIIME using the ‘most_abundant’ method. For bacteria, reference sequences of OTUs were assigned taxonomies using default arguments in the assign_taxonomy.py script in QIIME with the RDP method^28^. In the assign_taxonomy.py function, the most recent Greengenes database (ftp://greengenes.microbio.me/greengenes_release/gg_13_5/gg_13_8_otus.tar.gz) was used for OTU picking and taxonomic assignment. For microeukaryotes, reference sequences of OTUs were assigned taxonomies using the assign_taxonomy.py with the uclust method with a confidence threshold of 0.8. In the assign_taxonomy.py function, the PR2 database (http://ssu-rrna.org/pr2) was used^29^. We followed PR2 taxonomic descriptors (structured by the use of eight unique terms).

The make_otu_table.py script was used to produce two OTU by sample tables containing the abundance and taxonomic assignment of bacterial and microeukaryotic OTUs. A full description of sequence analysis can be found in supportive information. The tables were uploaded to R software (version 3.1.1; http://www.r-project.org/) for statistical computing and graphics and removal of unassigned and singleton OTUs, chloroplast and mitochondrial sequences.

Variation in composition among sites was assessed using principal coordinates analysis (PCO). The PCO was generated using the cmdscale() function in the R base package and wascores() function in vegan. Prior to the PCO, the raw data was log (x+1) transformed and used to produce a distance matrix with the Bray-Curtis index with the vegdist() function in vegan^30^. The procrustes() function in vegan was used to assess congruence among bacterial and microeukariotic PCO ordinations. Default values were used for the arguments in the procrustes() analysis. In addition to the procrustes() function, the protest() function in vegan was used to estimate the significance of the procrustes statistic. The number of permutations in the protest() function was set to 999. Pearson correlations between the most abundant bacterial orders (top 16) and microeukaryotic divisions (top 15) [log_e_ (x + 1) transformed] were computed using rcorr() from the Hmisc package^31^ and plotted using the corrplot R package^32^. The R vegan adonis() function for permutational multivariate analysis of variance (PERMANOVA) was used to test for significant variations in composition between SE M. Ivanov samples and all other locations. In the adonis analysis, the Bray-Curtis distance matrix of OTU composition was the response variable with samples as independent variables. The number of permutations was set at 999.

The closest relatives of the most abundant OTUs (≥50 sequences for bacteria and ≥200 sequences for microeukaryotes) were identified using the NCBI Basic Local Alignment Search Tool (BLAST) command line “blastn” tool with the -db argument set to nt^33^. We used the blastn command line tool to query representative sequences of selected taxa against the online NCBI nucleotide database. We then generated a vector containing sequence identifiers (GIs) of the ten top hits of all representative sequences and used the Entrez.efetch function in BioPython with the rettype argument set to ‘gb’ to download GenBank information including the isolation source of the organism. The list of bacterial, and microeukaryotic analysed OTUs can be found in Tables S1 and S2.

## Results

### Variation in bacterial community composition

**Figure 2:**
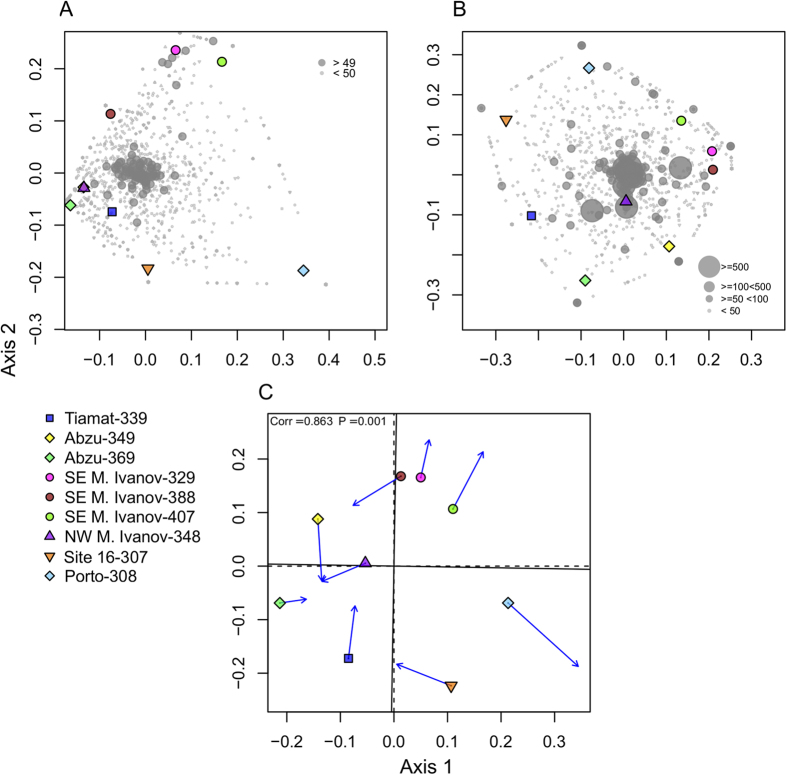
Ordination showing the first two axes of the PCO analysis for (**A**) bacterial OTU composition; (**B**) microeukaryotic OTU composition and (**C**) Procrustes analysis comparing bacterial (**A**) and microeukaryotic (**B**) OTU composition. In Procrustes analysis the arrows point to the target configuration (bacterial OTU composition), the symbols represent the rotated configuration (microeukaryotic composition). Correlation (Corr) and significance values (P) were calculated using the protest function from the vegan R package^30^. Numbers refer to OTU numbers in Tables S1 and S2.

The analysis of bacterial OTU composition revealed differences among samples collected from the various mud-volcanoes (Fig. 2A). Along the first axis of variation, sample 308 is clearly separated from the rest of the samples. This sample was collected in Porto MV, an active MV located on the AWGC. Along the second axis of variation, there was a strong separation between SE M. Ivanov MV samples (329, 388 and 407) and site 16 sample (307), with the remaining Horseshoe Valley samples occupying an intermediate position (Abzu -349, 369; Tiamat -339; NW M. Ivanov -348). Adonis analysis further revealed a significant difference in composition between SE M. Ivanov samples and the rest of the sampling sites (adonis: F_1,8_ = 1.424, R^2^ = 0.169, P = 0.037).

**Figure 3:**
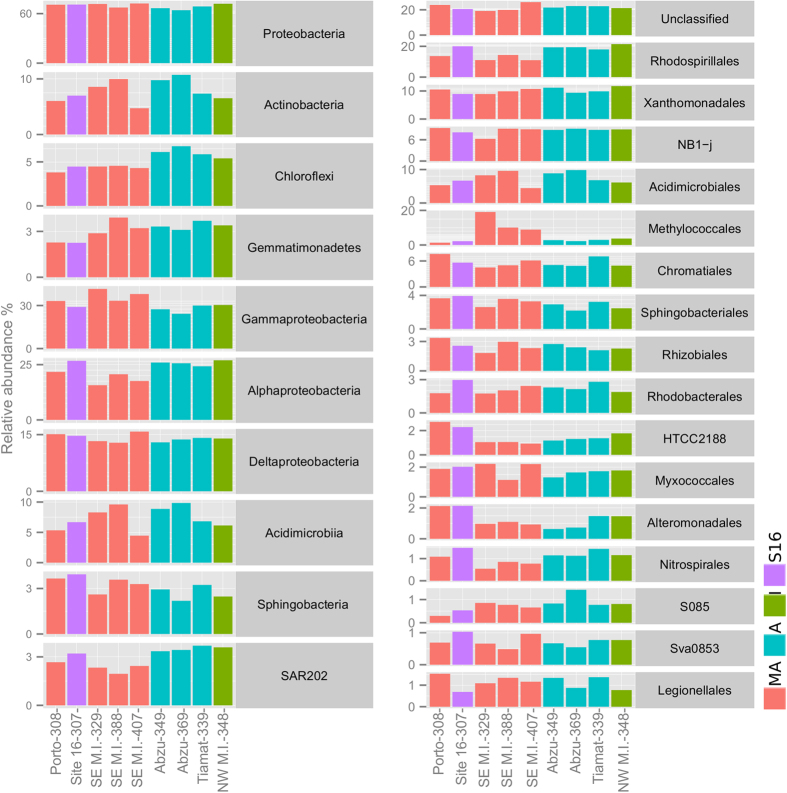
Relative abundance of the most abundant bacterial groups (four most abundant phyla, four most abundant classes, 16 most abundant orders and unclassified OTUs at order level). MA - Most active, A - active, I - inactive and S16 - site 16.

Using the RDP classifier tool with a confidence threshold of 80%, 23336 out of 23555 (99.07%) qualified bacterial sequences were assigned to known phyla. The number of qualified sequences varied from 1840 in Porto MV (308) to 2962 in Abzu MV (349). The most abundant phylum was *Proteobacteria* (average relative abundance of 69.39 ± 2.84%) followed by *Actinobacteria* (7.85 ± 2.02%), *Chloroflexi* (5.09 ± 0.99%) and *Gemmatimonadetes* (3.11 ± 0.57), comprising together 85.38% of all filtered sequences analysed (Fig. 3). Among these phyla, *Chloroflexi* had a lower relative abundance in SE M. Ivanov, Porto MV and site 16 (4.32 ± 0.11%) compared to the remaining samples (6.04 ± 0.57%). The most abundant bacterial classes were *Gammaproteobacteria* (31.90 ± 5.32%), *Alphaproteobacteria* (22.81 ± 4.11%), *Deltaproteobacteria* (14.06 ± 0.99%), *Acidimicrobiia* (7.34 ± 1.90), *Sphingobacteria* (3.01 ± 0.59) and SAR202 (2.96 ± 0.62). The relative abundance of *Alphaproteobacteria* was lower in the SE M. Ivanov MV (18.00 ± 2.49%) when compared to the other locations (25.22 ± 1.95%). This difference was mainly due to the order *Rhodospirillales* (12.41 ± 1.92% in SE M. Ivanov compared with 18.64 ± 2.69 in all other locations). Likewise, the SAR202 group also had a lower relative abundance in the SE M. Ivanov MV (2.24 ± 0.26%), when compared with the other locations (3.32 ± 0.36%). At the order level, there was a preponderance of *Methylococcales* in samples collected in the active area of SE M. Ivanov MV (average relative abundance of 12.69 ± 5.56%) when compared to other samples (2.56 ± 0.82%). Also at the order level, *Nitrospirales* were less abundant in SE M. Ivanov and Porto MV (0.82 ± 0.32%) when compared to the other locations (1.27 ± 0.18%).

The heatmap analysis (with dendrograms) (Figure S1) of the most abundant OTUs (≥50 sequences) confirmed the trend revealed by the PCO and the taxonomic analysis. The Porto MV sample (308), for example, contained a highly dissimilar bacterial community when compared to the other samples. Several OTUs assigned to the *Methylococcales* order (OTU 29, 18, 17, 135 and 700) were more abundant in samples collected from the SE M. Ivanov MV (329, 388 and 407) while several OTUs assigned to the *Rhodospirillales* order (OTU 47 and 32) and *Chloroflexi* phylum (OTUs 25, 31 and 43) were more abundant in the remaining locations.

### Variation in microeukaryotic community composition

The PCO analysis of microeukaryotic OTU composition revealed a similar pattern to that observed in the ordination analysis of the bacterial composition (Fig. 2B). Along the first axis SE M. Ivanov MV samples (329, 388 and 407) separated from site 16 sample (307), with the other Horseshoe Valley locations occupying an intermediate position. The site from Porto MV was separated from the Horseshoe Valley sites along the second axis. Most of the abundant OTUs clustered near the sample from the NW M. Ivanov MV (348). Adonis analysis also revealed a significant difference in composition between SE M. Ivanov samples and the rest of the samples (adonis: F_1,8_ = 1.424, R^2^ = 0.169, P = 0.037).

**Figure 4:**
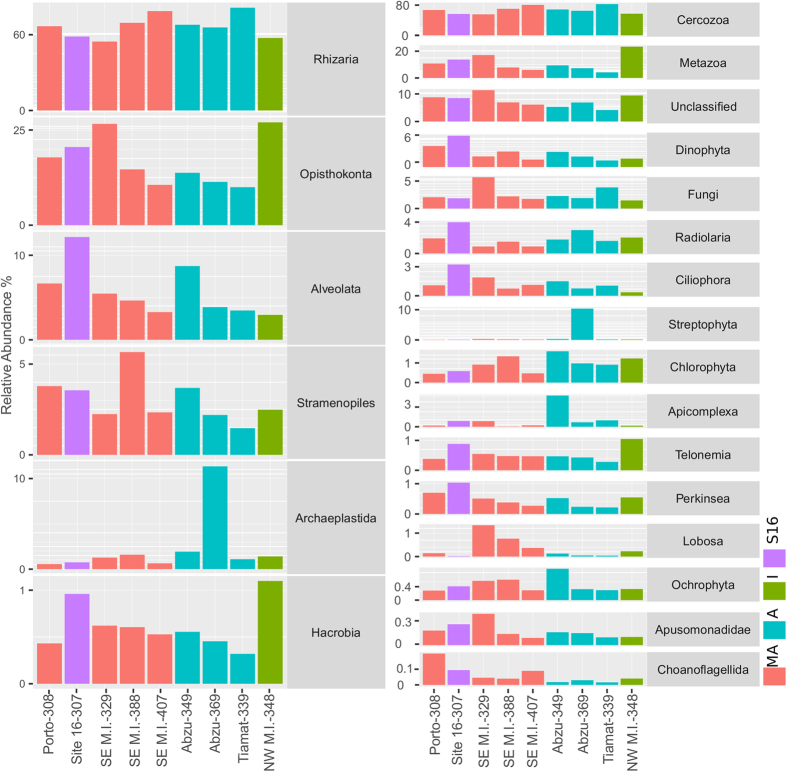
Relative abundance of the most abundant microeukaryotic groups (six most abundant phyla and 15 most abundant divisions and unclassified OTUs at division level). MA - Most active, A - active, I - inactive and S16 - site 16.

After classification and removal of unassigned and singleton OTUs, 53970 out of 55545 (97.16%) qualified sequences were assigned to known groups. The number of qualified sequences varied from 4003 in Abzu MV (369) to 7708 in Tiamat MV (339). At phylum level, *Rhizaria* was the most abundant (average relative abundance of 68.25 ± 8.74%), followed by *Opisthokonta* (17.33 ± 6.94%), *Alveolata* (5.62 ± 2.98%), *Stramenopiles* (3.04 ± 1.24%), *Archaeplastida* (3.59 ± 2.34%) and *Hacrobia* (0.61 ± 0.24%) (Fig. 4). Together, these six phyla comprised 99.51% of all filtered sequences analysed. The relative abundance of *Rhizaria*, the most abundant phylum, was slightly higher in samples 339 (82.12%), 407 (79.86%) and 388 (70.49%) than in the rest of the samples (63.63 ± 5.49%). This also held for the *Cercozoa* division. A high proportion of *Cercozoa* OTUs were assigned to *Endomyxa-Ascetosporea* (41.70 ± 12.95%). *Radiolaria* abundance was lower in SE M. Ivanov (1.01 ± 0.35%) than the other locations (2.38 ± 0.92%). In contrast, the *Lobosa* (lobose amoebae) division was more abundant in samples from SE M. Ivanov (0.82 ± 0.48%) when compared to the other samples (0.1 ± 0.07%).

The heatmap analysis (Figure S2) of the most abundant OTUs (≥200 sequences) separated four groups of sites: (i) one cluster grouped two sites from SE M. Ivanov and one from Abzu MV; (ii) the remaining Abzu and SE M. Ivanov MV sites clustered separately; (iii) the sample from Tiamat MV clustered with NW M. Ivanov and (iv) site 16 and Porto MV formed a fourth group. Samples from most sites housed OTUs that were restricted to that sample or were much less abundant in other sites (e.g., sample 369: OTUs 35 and 16; sample 329: OTUs 20 and 561; sample 349: OTUs 25 and 41; sample 348: OTUs 8 and 29). The relative abundances of some OTUs assigned to the *Ascetosporea* were higher in the active MVs when compared to inactive MVs and site 16 (e.g. OTUs 4, 2 and 18).

### Bacterial and microeukaryotic associations

In order to determine if there was any congruence between the trends observed from the bacterial and microeukaryotic communities, we compared the PCO ordinations obtained with both datasets using procrustes analysis. There was a significant congruence between both datasets (procrustes correlation, R = 0.863, P = 0.001; Fig. 2c). The correlation analysis between the most abundant bacterial orders and microeukaryotic divisions revealed several significant correlations (Figure S3). The strongest positive correlation was detected between *Methylococcales* and *Lobosa* (pearson correlation, R = 0.790, P = 0.011). Worthy of note, was also the correlation between *Rhodospirillales* and *Radiolaria* (pearson correlation, R = 0.721, P = 0.028)

## Discussion

### Bacterial community composition

In line with Pachiadaki & Kormas^34^, we assumed that the differences in seepage regime were likely the most important driver of compositional variation in the studied MVs. The relative dominance of proteobacterial OTUs in all locations was consistent with previous observations in deep-sea sediment^35^,^36^,^37^. Pachiadaki *et al*.^12^, also reported that the sediment bacterial community was dominated by the proteobacterial classes *Deltaproteobacteria*, *Gammaproteobacteria* and *Espilonproteobacteria* (23.1%, 22.3% and 14.9% respectively) in the Amsterdam MV (Mediterranean Sea). In our study, a higher proportion of *Alphaproteobacteria*, a group that was underrepresented in the Amsterdam MV, was detected. This class showed to be more abundant in active sites compared to non-active sites. This was mainly due to the variation in the abundance of the order *Rhodospirillales*, whose overall relative abundance was higher (16.43%) when compared to values reported from other studies^12^. This order includes two families: the *Acetobacteraceae* and the *Rhodospirillaceae*^38^. A high proportion of OTUs detected in this study were assigned to the *Rhodospirillaceae* that was initially described as a group of anaerobic photosynthetic bacteria and later, through phylogenetic similarity, as non-photosynthetic aerobic/microaerobic bacteria^39^. Interestingly, many novel isolates classified to *Rhodospirillaceae* retrieved from the deep-sea are associated with petroleum hydrocarbon degradation^40^,^41^,^42^. However, BLAST similarity search of the most predominant *Rhodospirillaceae* OTUs did not reveal similarity with sequences found in oil-impacted environments (Table S1). In line with the variations in the relative abundance observed for *Rhodospirillaceae*, the *Nitrospirales* order was less abundant in the most active sites (SE M. Ivanov). The *Nitrospirales* order only consists of the family *Nitrospiraceae*, whose members are physiologically diverse and include aerobic nitrate oxidizers, aerobic and acidophilic iron oxidizers and hydrogenotrophic sulfate reducers^43^. In a comparison of active and non-active vent bacteria, Cerqueira *et al*.^44^ found a similar pattern to the one observed in this study.

Several of the most abundant *Rhodospirillaceae* and the main *Nitrospirales* OTUs were similar to sequences retrieved from deep-sea manganese-iron nodules (Table S1, OTUs 3, 10, 16, 30, 32, 39 and 56). Manganese-iron nodules are thought to develop through the combined action of abiotic and biotic processes in deep-sea sediments with low sedimentation rates and are consequently poor in organic carbon^45^. Manganese oxide is a more favourable electron acceptor than iron oxide or sulphate and, therefore, manganese-reducing microorganisms can outcompete iron and sulphate reducers^46^. Manganese-reduction could be an important metabolic alternative for bacterial communities in the less active MVs. SAR202 class also displayed a similar trend to *Rhodospirillaceae* and *Nitrospirales.* This class is ubiquitous among microbiota of meso- and bathyalpelagic zones and is known to increase in abundance with depth, denoting adaptation to oligotrophic conditions^47^,^48^,^49^. The ecological niche occupied by SAR202 is still not fully understood; nonetheless SAR202 has been characterized as an r-strategist and is thought to play an important role in dissolved organic matter recycling in the deep ocean^47^. The order HTCC2188 was less abundant in M. Ivanov active sites when compared to other sites (Fig. 3). As is the case with SAR202, the order HTCC2188 has also been characterized as oligotrophic^50^. In contrast to these taxa, the relative abundance of *Methylococcales* was higher in active areas of SE M. Ivanov MV. This was not surprising, considering that the most distinct characteristic of this group is the ability to use methane as a sole carbon and energy source^51^. OTUs that clustered near the active area of M. Ivanov MV were mainly assigned within this order and had high similarity to sequences retrieved from other methane rich environments namely, hydrothermal vents [OTU 18 and 29^52^ and OTU 700^53^] and MVs [OTU 17 and OTU 135^13^]. At the active Porto MV site, the relative abundance of *Methylococcales* was lower than at the M. Ivanov active sites. However, an abundant OTU, assigned to the *Methylococcaceae* family (OTU 102), was retrieved from the active Porto MV, thus underlining the importance of this group in active MVs. This trend was clearer in the heatmap analysis (Figure S1) that clustered the active MV sites (M. Ivanov and Porto) based on the composition of the most abundant OTUs (≥50 sequences).

Overall, the spatial distributions of bacterial communities studied here showed similar trends to those observed by Cerqueira *et al*.^44^ in a hydrothermal vent^44^. Cerqueira *et al*.^44^ found that the relative abundance of *Methylococcales* in the proximity of a vent chimney complex was significantly higher than in the abyssal plain surrounding the complex, while the reverse was true for *Rhodospirillales* and *Nitrospirales*. The *Deltaproteobacteria* only showed minor variation in abundance among the sampling sites. This class includes sulfate-reducers, that are usually abundant in MVs and are often identified as one of the key functional groups in these ecosystems^34^. At the order level, most of the deltaproteobacterial OTUs were assigned to uncultivated groups (e.g. NB1-j and Sva0853). The NB-j taxa groups uncultured related sequences and their function in the marine microbial community is unknown. Nonetheless, it is possible that this group may be involved in the process of hydrocarbon degradation. Mason *et al*.^54^ showed that bacterial members of the NB1-j groups represented 9.65% of RNA sequences in samples collected near the Deep Water Horizon accident site and were less abundant in more distant sites and the control site. However, in our study NB1-j was abundant in all sites with no evidence of a significant effect of MV activity on the compositional distribution of this group.

### Microeukaryotic community composition

The microeukaryote community composition was dominated by OTUs assigned to the *Endomyxa-Ascetosporea* (*Rhizaria* phylum, *Cercozoa* division) in all samples. Although the *Cercozoa* group has been shown to increase with depth and had the highest relative abundance in deep-sea hydrothermal vent samples^55^, it is not commonly revealed by metagenomic studies as dominant in deep-sea sediments. The phylum *Alveolata* is usually found in deep-sea sediment surveys as the most abundant^56^,^57^,^58^,^59^,^60^, while *Fungi*, *Stramenophiles* and *Flabellinea* dominance has also been reported^55^,^59^.

The *Radiolaria* division (*Rhizaria* phylum) was less abundant in active MVs than in other sites. This division is part of the *Rhizaria* phylum, that besides *Radiolaria* also includes the *Foraminifera*^61^. The low *Foraminifera* abundance in methane vents and other methane enriched environments had previously been reported, presumably due to extreme pCO_2_ levels^62^. *Radiolaria* share several features with *Foraminifera*, namely the formation of tests. In contrast, the *Lobosa* division was more abundant in samples from the active SE M. Ivanov crater. This group includes non-flagellate lobose amoebae and is partitioned into the classes *Tubulinea* (tube-shaped pseudopodia) and *Discosea* (flattened cells)^63^.

### Bacteria and microeukaryotic associations

There was a significant congruence between bacterial and the microeukaryotic datasets, as revealed by the Procrustes analysis. Co-occurrence patterns might result either from abiotic processes, that act independently on each taxon, or from interactions such as metabolic interdependence, facilitation or predation^64^. This combined response was clear in the active area of M. Ivanov, which displayed a consistent presence of OTUs assigned to the *Methylococcae* and *Lobosa*. This relationship was apparent in the variation of relative abundances of *Methycoccolales* and *Lobosa* among samples and further confirmed by the correlation analysis between the most abundant bacterial orders and microeukaryotic divisions (Figure S3). Although our analysis did not resolve the nature of the correlation, previous studies suggest that a prey-predator relation could be the basis of the co-occurrence between *Methycoccolales* and *Lobosa.* For example, Murase and Frenzel^65^ provided evidence that lobose amoebae inhabiting rice field sediments are important grazers of bacterial methanotrophs^65^. Likewise, Pernice *et al*.^66^ recently found a good correlation of heterotrophic protists with prokaryotic abundance that suggested active grazing of protists on prokaryotes in deepwater samples^66^. Such a relationship could indicate an important role of members of the *Lobosa* division as first-level consumers of bacterial methanotrophs and subsequent transfer of methane metabolic energy to higher trophic levels in active MVs.

On the other hand, both *Rhodospirillales* and *Radiolaria* were less abundant in SE M. Ivanov and Porto MVs. The BLAST analysis appears to support an association between *Rhodospiralles* and members of the *Rhizaria* phylum; two of the most abundant *Rhodospirillales* OTUs (19 and 22) were highly similar (sequences similarity = 99%) to OTUs from deep-sea sediment surrounding colonies of giant foraminifera at the Pacific Ocean (*Xenophyophorea*)^67^.

## Conclusions

There was significant congruence between bacterial and microeukaryotic composition in sampling sites with different seepage regimes. Within the bacterial communities and at the order level, *Methylococcales* were more abundant and *Rhodospirillales*, *Nitrospirales* and SAR202 less abundant in the most active sites. Within the microeukaryotic communities, the *Radiolaria* group were less abundant and lobose amoebae (putative methanotrophic bacteria grazer) more abundant in the most active MVs. The strong correlation between the relative abundance of *Methylococcales* and lobose amoebae in active MVs raises the possibility that the *Lobosa* division may play an important role as first-level consumers in the incorporation of methane-derived carbon into eukaryotic biomass. Such function is generally associated with bacteriovorous ciliates in the deep-sea environment^8^. However, this study cannot determine the nature of this correlation and although a prey-predator relationship appears to be the most plausible hyphotesis, other mechanisms including co-colonization or co-survival of the same habitat by the two groups can also explain this pattern^68^. Further studies are necessary to investigate the ecological relationship between members of the lobose amoebae and methanotrophic bacterial communities in deep-sea MVs.

## Additional Information

**How to cite this article**: Coelho, F. J. R. C. *et al*. Integrated analysis of bacterial and microeukaryotic communities from differentially active mud volcanoes in the Gulf of Cadiz. *Sci. Rep.* **6**, 35272; doi: 10.1038/srep35272 (2016).

## Supplementary Material

Supplementary Information

## Figures and Tables

**Table 1:** Description of the sampling stations.

	Location	St.	Date dd.mm.yy	Gear	Latitude (N)	Longitude (W)	Depth (m)
AWGC	Porto MV	308	27.02.12	MUC	35°33.73′	9°30.46′	3909
HV	SE M. Ivanov MV	329	01.03.12	BC	35°44.33′	10°12.05′	4492
		388	11.03.12	BC	35°44.33′	10°12.07′	4485
		407	14.03.12	BC	35°44.34′	10°12.05′	4507
HV	NW M. Ivanov MV	348	05.03.12	BC	35°44.41′	10°12.18′	4497
HV	Tiamat MV	339	03.03.12	BC	35°45.78′	10°21.33′	4551
HV	Abzu MV	349	05.03.12	BC	35°45.05′	10°19.04′	4560
		369	08.03.12	BC	35°45.04′	10°19.03′	4550
HV	Site 16	307	27.02.12	MUC	35°42.00′	9°57.92′	4585

St. Station number; AWGC: Accretionary wedge of the Gulf of Cadiz; HV: Horseshoe Valley; BC: boxcorer; MUC: Multicorer.
