# Supplementary material for: Integrated analysis of bacterial and microeukaryotic communities from differentially active mud volcanoes in the Gulf of Cadiz
Source: Sci Rep. 2016 Oct 20;6:35272. doi: 10.1038/srep35272 (PMC5071872; doi:10.1038/srep35272)
Supplement: Supplementary Information [file srep35272-s1.doc]

Supplementary information

Integrated analysis of bacterial and microeukaryotic communities from differentially active mud volcanoes in the Gulf of Cadiz

Francisco J.R.C. Coelho^1^, António Louvado^1^, Patrícia M. Domingues^1,2^, Daniel Cleary^1^, Marina Ferreira^1^, Adelaide Almeida^1^, Marina R. Cunha^1^, Ângela Cunha^1^ and Newton C. M. Gomes^1^*.

^1^Department of Biology & CESAM, University of Aveiro, Campus de Santiago, 3810-193 Aveiro, Portugal

^2^Department of Chemistry & CICECO, University of Aveiro, Campus de Santiago, 3810-193 Aveiro, Portugal

Sequence analysis of the 16S and 18S rRNA gene fragments

Sequencing analysis was performed using QIIME software (Quantitative Insights into Microbial Ecology - http://www.qiime.org/; version 1.8.0) ^1^ following previously described methods ^2^. In QIIME, fasta and qual files were used as input for the split_libraries.py script. Default arguments were used except for the minimum sequence length, which was set at 218 bps after removal of forward primers and barcodes; reverse primers were removed using the 'truncate only' argument and a sliding window test of quality scores was enabled with a value of 50 as suggested in the QIIME description for the script. In addition to user-defined cutoffs, the split_libraries.py script performs several quality filtering steps (http://qiime.org/scripts/split_libraries.html). OTUs were selected using UPARSE with usearch7 ^3^. The UPARSE sequence analysis tool provides clustering, chimera checking and quality filtering on de-multiplexed sequences. Chimera checking was performed using the UCHIME algorithm. First reads were filtered with the -fastq_filter command and the following arguments -fastq_trunclen 250 -fastq_maxee 0.5 -fastq_truncqual 15. Sequences were then dereplicated and sorted using the -derep_fulllength and - sortbysize commands. OTU clustering was performed using the -cluster_otus command (cut-off threshold at 97%). An additional chimera check was subsequently applied using the -uchime_ref command with the gold.fa database (http://drive5.com/uchime/gold.fa). AWK scripts were then used to convert the otus to QIIME format. In QIIME, representative sequences were selected using the pick_rep_set.py script in QIIME using the 'most_abundant' method. For bacteria, reference sequences of OTUs were assigned taxonomies using default arguments in the assign_taxonomy.py script in QIIME with the rdp method ^4^. In the assign_taxonomy.py function, the most recent Greengenes database (ftp://greengenes.microbio.me/greengenes_release/gg_13_5/gg_13_8_otus.tar.gz) was used for OTU picking and taxonomic assignment. For microeukaryotes, reference sequences of OTUs were assigned taxonomies using the assign_taxonomy.py with the uclust method with a confidence threshold of 0.8. In the assign_taxonomy.py function, the PR2 database (http://ssu-rrna.org/pr2) was used for taxonomic assignment ^5^. The make_otu_table.py script was used to produce two OTU by sample tables containing the abundance and taxonomic assignment of bacterial and microeukaryotic OTUs. This was subsequently used as input for further analyses using the R package (R Core Team 2013).

Figure S1: Heatmap showing the abundance of dominant 16S rRNA sequence reads (≥ 50 sequences). The heatmap was generated using the function heatmap2() in the R package gplots (http://www.cran.r-project.org/). The OTU's were log-transformed and clustered according to their occurrence by UPGMA hierarchical clustering.

Figure S2: Heatmap showing the abundance of dominant 18S rRNA sequence reads (≥ 200 sequences). The heatmap was generated using the function heatmap2() in the R package gplots (http://www.cran.r-project.org/). The OTU's were log-transformed and clustered according to their occurrence by UPGMA hierarchical clustering.

Figure S3: Correlation matrix based on Pearson's Rank correlation coefficients between most abundant bacterial orders (top 16) and microeukaryotic divisions (top 15). The size and intensity of color for each square represents the strength of the correlation (the larger, darker squares demonstrate a strong correlation); blue colors illustrate positive correlations and red colors illustrate negative correlation coefficients. Correlations that are significant (P < 0.05) are marked with an x.

Table S1 – List of most abundant bacterial OTUs (≥ 50 sequences). The table includes the OTU-numbers (OTU); taxonomic assignment generated by QIIME; GenInfo Identifier (GI), sequence similarity of the closest matches with our representative OTU sequences (Seq. Sim.) and the source of these organisms (Source/Context).

| **OTU** | **Phylum** | **Class** | **Order** | **Family** | **Genus** | **GI** | **Seq. Sim** | **Source/Context** |
| --- | --- | --- | --- | --- | --- | --- | --- | --- |
| 1 | Actinobacteria | Acidimicrobiia | Acidimicrobiales | koll13 | Unclassified | 730046669 | 100 | marine sediment, Maluku Strait |
| 4 | Proteobacteria | Alphaproteobacteria | Rhodospirillales | Rhodospirillaceae | Unclassified | 459465879 | 100 | deep-sea polymetallic nodule, Clarion-Clipperton Fracture Zone |
| 2 | Proteobacteria | Gammaproteobacteria | Xanthomonadales | Unclassified | Unclassified | 910280221 | 100 | Deep-sea manganese nodule |
| 3 | Proteobacteria | Alphaproteobacteria | Rhodospirillales | Rhodospirillaceae | Unclassified | 910280217 | 100 | Deep-sea manganese nodule |
| 6 | Proteobacteria | Gammaproteobacteria | Xanthomonadales | Unclassified | Unclassified | 910280227 | 100 | Deep-sea manganese nodule |
| 7 | Proteobacteria | Gammaproteobacteria | Unclassified | Unclassified | Unclassified | 347823799 | 100 | Deep sea surface sediment from Indian Ocean |
| 8 | Proteobacteria | Gammaproteobacteria | Methylococcales | Methylococcaceae | Unclassified | 910280249 | 100 | Deep-sea manganese nodule |
| 9 | Proteobacteria | Alphaproteobacteria | Rhodobacterales | Rhodobacteraceae | Unclassified | 459465277 | 100 | deep-sea polymetallic nodule, Clarion-Clipperton Fracture Zone |
| 10 | Proteobacteria | Alphaproteobacteria | Rhodospirillales | Rhodospirillaceae | Unclassified | 459465729 | 100 | deep-sea polymetallic nodule, Clarion-Clipperton Fracture Zone |
| 11 | Proteobacteria | Gammaproteobacteria | Xanthomonadales | Unclassified | Unclassified | 459465698 | 100 | deep-sea polymetallic nodule, Clarion-Clipperton Fracture Zone |
| 12 | Proteobacteria | Gammaproteobacteria | Methylococcales | Methylococcaceae | Unclassified | 459466002 | 100 | deep-sea polymetallic nodule, Clarion-Clipperton Fracture Zone |
| 13 | Proteobacteria | Gammaproteobacteria | Xanthomonadales | Unclassified | Unclassified | 910280209 | 100 | Deep-sea manganese nodule |
| 14 | Proteobacteria | Gammaproteobacteria | Chromatiales | Unclassified | Unclassified | 459465834 | 100 | deep-sea polymetallic nodule, Clarion-Clipperton Fracture Zone |
| 15 | Proteobacteria | Alphaproteobacteria | Rhodospirillales | Rhodospirillaceae | Unclassified | 459465645 | 100 | seamount sediments in the Pacific Ocean |
| 16 | Proteobacteria | Alphaproteobacteria | Rhodospirillales | Rhodospirillaceae | Unclassified | 910280279 | 100 | Deep-sea manganese nodule |
| 17 | Proteobacteria | Gammaproteobacteria | Methylococcales | Unclassified | Unclassified | 224713869 | 99.53 | sediments of an active submarine mud volcano, Kazan Mud Volcano, Anaximander Mountains, East Mediterranean Sea |
| 18 | Proteobacteria | Gammaproteobacteria | Methylococcales | Unclassified | Unclassified | 756983378 | 99.53 | ventral setae of deep-sea crab Shinkaia crosnieri |
| 19 | Proteobacteria | Alphaproteobacteria | Rhodospirillales | Rhodospirillaceae | Unclassified | 392305965 | 99.75 | Deep-sea sediment with and without xenophyophore at a depth 7111m |
| 20 | Proteobacteria | Gammaproteobacteria | Chromatiales | Unclassified | Unclassified | 459465141 | 99.77 | deep-sea polymetallic nodule, Clarion-Clipperton Fracture Zone |
| 21 | Gemmatimonadetes | Gemm-4 | Unclassified | Unclassified | Unclassified | 459465960 | 99.77 | deep-sea polymetallic nodule, Clarion-Clipperton Fracture Zone |
| 22 | Proteobacteria | Alphaproteobacteria | Rhodospirillales | Rhodospirillaceae | Unclassified | 392305978 | 99.26 | Deep-sea sediment with and without xenophyophore at a depth 7111m |
| 23 | Proteobacteria | Gammaproteobacteria | Chromatiales | Unclassified | Unclassified | 459465708 | 100 | seamount sediments in the Pacific Ocean |
| 24 | Proteobacteria | Deltaproteobacteria | NB1-j | NB1-i | Unclassified | 730046682 | 100 | marine sediment, Maluku Strait |
| 25 | Chloroflexi | S085 | Unclassified | Unclassified | Unclassified | 864422637 | 100 | hadopelagic sediments in the Challenger Deep, Mariana Trench |
| 26 | Chloroflexi | SAR202 | Unclassified | Unclassified | Unclassified | 283475783 | 100 | deep-sea surface sediments of the South Atlantic Ocean, Guinea Basin |
| 27 | Proteobacteria | Gammaproteobacteria | Unclassified | Unclassified | Unclassified | 270282792 | 94.88 | marine sediments |
| 29 | Proteobacteria | Gammaproteobacteria | Methylococcales | Unclassified | Unclassified | 756983074 | 97.44 | bacterial community exposed to intestinal extract of deep-sea crab Shinkaia crosnieri |
| 30 | Proteobacteria | Alphaproteobacteria | Rhodospirillales | Rhodospirillaceae | Unclassified | 350542336 | 98.77 | manganese oxide-rich marine surface sediment |
| 31 | Chloroflexi | TK17 | S085 | Unclassified | Unclassified | 291066055 | 100 | deep-sea sediments of polymetallic nodule province |
| 32 | Proteobacteria | Alphaproteobacteria | Rhodospirillales | Rhodospirillaceae | Unclassified | 459465824 | 100 | deep-sea polymetallic nodule, Clarion-Clipperton Fracture Zone |
| 33 | Proteobacteria | Alphaproteobacteria | Rhizobiales | Hyphomicrobiaceae | Hyphomicrobium | 927820290 | 100 | seafloor basalt, 3 km depth |
| 34 | Actinobacteria | Acidimicrobiia | Acidimicrobiales | koll13 | Unclassified | 392306286 | 100 | Deep-sea sediment with and without xenophyophore at a depth 7111m |
| 35 | Actinobacteria | Acidimicrobiia | Acidimicrobiales | koll13 | Unclassified | 290752207 | 100 | cutaneous mucus of wild seahorses |
| 36 | Proteobacteria | Gammaproteobacteria | Chromatiales | Unclassified | Unclassified | 219809896 | 100 | deep marine sediments from hydrothermal region, at 2725 m depth |
| 37 | Proteobacteria | Alphaproteobacteria | Rhodospirillales | Rhodospirillaceae | Unclassified | 316980834 | 100 | hadopelagic sediments in the Ogasawara Trench |
| 38 | Proteobacteria | Gammaproteobacteria | HTCC2188 | HTCC2089 | Unclassified | 46560359 | 98.55 | oxic surface sediments of eastern Mediterranean Sea |
| 39 | Nitrospirae | Nitrospira | Nitrospirales | Nitrospiraceae | Unclassified | 910280228 | 100 | Deep-Sea Manganese Nodules |
| 40 | Nitrospirae | Nitrospira | Nitrospirales | Nitrospiraceae | Unclassified | 459465937 | 100 | deep-sea polymetallic nodule, Clarion-Clipperton Fracture Zone |
| 41 | Proteobacteria | Deltaproteobacteria | NB1-j | NB1-i | Unclassified | 459465052 | 100 | deep-sea polymetallic nodule, Clarion-Clipperton Fracture Zone |
| 42 | Actinobacteria | Acidimicrobiia | Acidimicrobiales | wb1_P06 | Unclassified | 169907948 | 100 | seafloor lavas from the East Pacific Rise |
| 43 | Chloroflexi | SAR202 | Unclassified | Unclassified | Unclassified | 752841779 | 100 | hydrothermal plumes |
| 45 | Proteobacteria | Gammaproteobacteria | HTCC2188 | HTCC2089 | Unclassified | 283476201 | 100 | Deep-sea sediment |
| 47 | Proteobacteria | Alphaproteobacteria | Rhodospirillales | Rhodospirillaceae | Unclassified | 169908021 | 100 | Seafloor lavas from the East Pacific Rise |
| 49 | Proteobacteria | Alphaproteobacteria | Rhizobiales | Hyphomicrobiaceae | Unclassified | 524452153 | 100 | deep-sea methane seep sediment |
| 50 | Gemmatimonadetes | Gemm-4 | Unclassified | Unclassified | Unclassified | 322367670 | 99.77 | marine sediments at the southern edge of the South Pacific Gyre |
| 51 | Proteobacteria | Alphaproteobacteria | Rhodobacterales | Rhodobacteraceae | Unclassified | 673524003 | 99.51 | deep-sea coral community impacted by the Deepwater Horizon oil spil |
| 53 | Proteobacteria | Alphaproteobacteria | Rhodospirillales | Rhodospirillaceae | Unclassified | 165882710 | 99.26 | 13 m depth in the Pacific Ocean |
| 55 | Proteobacteria | Deltaproteobacteria | NB1-j | NB1-i | Unclassified | 459465256 | 100 | deep-sea polymetallic nodule, Clarion-Clipperton Fracture Zone |
| 56 | Nitrospirae | Nitrospira | Nitrospirales | Nitrospiraceae | Unclassified | 910280280 | 100 | Deep-sea manganese nodule |
| 57 | Firmicutes | Bacilli | Unclassified | Unclassified | Unclassified | 820983273 | 100 | soil, Mediterranean |
| 62 | Proteobacteria | Alphaproteobacteria | Rhodospirillales | Rhodospirillaceae | Unclassified | 334303169 | 100 | sediments of Medea hypersaline basin, Mediterranean Sea |
| 63 | Chlorobi | Ignavibacteria | Ignavibacteriales | Ignavibacteriaceae | Unclassified | 283475501 | 100 | deep-sea surface sediments of the South Atlantic Ocean |
| 65 | Gemmatimonadetes | Gemm-1 | Unclassified | Unclassified | Unclassified | 768028330 | 99.51 | coral Porites lutea |
| 68 | Proteobacteria | Gammaproteobacteria | Chromatiales | Unclassified | Unclassified | 71148514 | 99.77 | basalt glass from 9N latitude East Pacific Rise |
| 69 | Proteobacteria | Deltaproteobacteria | Sva0853 | JTB36 | Unclassified | 730046583 | 100 | marine sediment, Maluku Strait |
| 71 | Actinobacteria | Acidimicrobiia | Acidimicrobiales | koll13 | Unclassified | 364536998 | 100 | deep-sea methane seeps at hikurangi continental margin, new zealand |
| 73 | Proteobacteria | Gammaproteobacteria | Xanthomonadales | Unclassified | Unclassified | 348590918 | 100 | surface sediments of an ultramafic hydrothermal vent field |
| 74 | Proteobacteria | Alphaproteobacteria | Rhodospirillales | Rhodospirillaceae | Unclassified | 347823768 | 99.75 | deep-sea sediments from the Indian Ocean |
| 77 | Chloroflexi | SAR202 | Unclassified | Unclassified | Unclassified | 342357474 | 99.01 | ocean wate, continental slope off Cape Lookout, NC from 505m depth |
| 78 | Proteobacteria | Gammaproteobacteria | Chromatiales | Unclassified | Unclassified | 472442811 | 99.7 | basaltic andesite, Mariner vent field, Lau Basin |
| 83 | Acidobacteria | Acidobacteria-6 | CCU21 | Unclassified | Unclassified | 459465905 | 100 | deep-sea polymetallic nodule, Clarion-Clipperton Fracture Zone |
| 85 | Proteobacteria | Gammaproteobacteria | Alteromonadales | OM60 | Unclassified | 283476185 | 99.3 | deep-sea surface sediments of the South Atlantic Ocean, Guinea Basin |
| 86 | Proteobacteria | Alphaproteobacteria | Unclassified | Unclassified | Unclassified | 459465232 | 99.75 | deep-sea polymetallic nodule, Clarion-Clipperton Fracture Zone |
| 88 | Proteobacteria | Deltaproteobacteria | NB1-j | JTB38 | Unclassified | 673524031 | 99.53 | deep-sea coral community impacted by the Deepwater Horizon oil spil |
| 89 | Proteobacteria | Alphaproteobacteria | Rhodobacterales | Rhodobacteraceae | Thalassobius | 459465316 | 100 | deep-sea polymetallic nodule, Clarion-Clipperton Fracture Zone |
| 93 | Bacteroidetes | Sphingobacteriia | Sphingobacteriales | Ekhidnaceae | Unclassified | 392306122 | 99.76 | Deep-sea sediment with and without xenophyophore at a depth 7111m |
| 94 | Gemmatimonadetes | Gemm-1 | Unclassified | Unclassified | Unclassified | 347823779 | 100 | deep-sea sediments from the Indian Ocean |
| 95 | Proteobacteria | Gammaproteobacteria | Unclassified | Unclassified | Unclassified | 392306339 | 100 | Deep-sea sediment with and without xenophyophore at a depth 7111m |
| 102 | Proteobacteria | Gammaproteobacteria | Methylococcales | Methylococcaceae | Unclassified | 364536898 | 97.9 | deep-sea methane seeps at hikurangi continental margin, new zealand |
| 104 | Proteobacteria | Gammaproteobacteria | Unclassified | Unclassified | Unclassified | 219809903 | 99.07 | deep marine sediments from hydrothermal region, at 2725 m depth |
| 105 | Proteobacteria | Alphaproteobacteria | Rhodospirillales | Rhodospirillaceae | Unclassified | 283475374 | 100 | deep-sea surface sediments of the South Atlantic Ocean |
| 109 | Proteobacteria | Deltaproteobacteria | NB1-j | MND4 | Unclassified | 459465882 | 98.84 | deep-sea polymetallic nodule, Clarion-Clipperton Fracture Zone |
| 113 | Proteobacteria | Gammaproteobacteria | Xanthomonadales | Unclassified | Unclassified | 283475353 | 100 | deep-sea surface sediments of the South Atlantic Ocean |
| 114 | Proteobacteria | Deltaproteobacteria | NB1-j | NB1-i | Unclassified | 392306350 | 99.54 | Deep-sea sediment with and without xenophyophore at a depth 7111m |
| 118 | Actinobacteria | Acidimicrobiia | Acidimicrobiales | koll13 | Unclassified | 459465046 | 100 | seamount sediments in the Pacific Ocean |
| 129 | Proteobacteria | Betaproteobacteria | Unclassified | Unclassified | Unclassified | 378405778 | 99.77 | low temperature hydrothermal oxides at the South West Indian Ridge |
| 133 | Proteobacteria | Gammaproteobacteria | Xanthomonadales | Unclassified | Unclassified | 472442703 | 99.77 | basalt, ABE vent field, Lau Basin |
| 135 | Proteobacteria | Gammaproteobacteria | Methylococcales | Unclassified | Unclassified | 224714027 | 99.3 | sediments of an active submarine mud volcano, Kazan Mud Volcano, Anaximander Mountains, East Mediterranean Sea |
| 191 | Proteobacteria | Gammaproteobacteria | Chromatiales | Unclassified | Unclassified | 283105773 | 99.77 | deep-sea ocean, 700-800 depth from sea surface |
| 239 | Proteobacteria | Gammaproteobacteria | Xanthomonadales | Unclassified | Unclassified | 910280210 | 100 | Deep-Sea Manganese Nodules |
| 266 | Proteobacteria | Gammaproteobacteria | Chromatiales | Unclassified | Unclassified | 406821826 | 99.77 | highly copper-contaminated marine sediment |
| 642 | Proteobacteria | Gammaproteobacteria | Xanthomonadales | Unclassified | Unclassified | 389592250 | 99.77 | subseafloor sediment at the Deformation Front |
| 700 | Proteobacteria | Gammaproteobacteria | Methylococcales | Methylococcaceae | Unclassified | 154347010 | 98.83 | hydrothermal sediments, Southern Okinawa trough |
| 820 | Proteobacteria | Gammaproteobacteria | Xanthomonadales | Unclassified | Unclassified | 459465676 | 100 | seamount sediments in the Pacific Ocean |
| 845 | Actinobacteria | Acidimicrobiia | Acidimicrobiales | koll13 | Unclassified | 50830636 | 99.75 | deep-sea mud volcanoes in the eastern Mediterranean |
| 2208 | Proteobacteria | Gammaproteobacteria | Xanthomonadales | Unclassified | Unclassified | 459465211 | 100 | deep-sea polymetallic nodule, Clarion-Clipperton Fracture Zone |
| 2476 | Proteobacteria | Gammaproteobacteria | Xanthomonadales | Unclassified | Unclassified | 348590890 | 99.77 | oceanic sediment, Logatchev hydrothermal vent field |
| 2580 | Proteobacteria | Gammaproteobacteria | Xanthomonadales | Unclassified | Unclassified | 459465470 | 99.77 | deep-sea polymetallic nodule, Clarion-Clipperton Fracture Zone |
| 3212 | Proteobacteria | Alphaproteobacteria | Rhizobiales | Hyphomicrobiaceae | Hyphomicrobium | 844683732 | 100 | Costa Rica mud volcano sediment |

Table S2 – List of most abundant microeukaryote (≥ 200 sequences). The table includes the OTU-numbers (OTU); taxonomic assignment generated by QIIME; GenInfo Identifier (GI), sequence similarity of the closest matches with our representative OTU sequences (Seq. Sim.) and the source of these organisms (Source/Context).

| **OTU** | **Phylum** | **Division** | **Class** | **Order** | **Family** | **Genus** | **GI** | **Seq. Sim** | **Source/Context** |
| --- | --- | --- | --- | --- | --- | --- | --- | --- | --- |
| 1 | Rhizaria | Cercozoa | Endomyxa-Ascetosporea | Paradinida | Paradinida | Paradinida | 225420930 | 92.05 | Eastern Mediterranean sapropels |
| 2 | Rhizaria | Cercozoa | Endomyxa-Ascetosporea | Paradinida | Paradinida | Paradinida | 225420930 | 92.88 | Eastern Mediterranean sapropels |
| 4 | Rhizaria | Cercozoa | Endomyxa-Ascetosporea | Paradinida | Paradinidae | Paradinium | 95115876 | 98.22 | hydrothermal field, Lost City |
| 5 | Rhizaria | Cercozoa | Filosa-Thecofilosea | Ventricleftida | CCW10-lineage | CCW10-lineage | 156454071 | 98.64 | sediment from the Kings Bay, Svalbard,Arctic |
| 6 | Opisthokonta | Unassigned | Unassigned | Unassigned | Unassigned | Unassigned | 636658184 | 96.74 | East Pacific Rise, 2500m |
| 7 | Rhizaria | Cercozoa | Filosa-Thecofilosea | Cryomonadida | Protaspa-lineage | Protaspa-lineage | 307149435 | 99.73 | Coastal surface waters, Chile |
| 8 | Opisthokonta | Metazoa | Platyhelminthes | Turbellaria | Acoelomorpha | Pseudaphanostoma | 378780946 | 90.08 | - |
| 11 | Rhizaria | Cercozoa | Endomyxa-Ascetosporea | Paradinida | Paradinidae | Paradinium | 95115876 | 99.70 | Lost City hydrothermal field |
| 12 | Rhizaria | Cercozoa | Endomyxa-Ascetosporea | Paradinida | Paradinida | Paradinida | 194272484 | 94.89 | Rhizobium |
| 13 | Archaeplastida | Chlorophyta | Unassigned | Unassigned | Unassigned | Unassigned | 672352215 | 94.20 | fresh water |
| 14 | Rhizaria | Cercozoa | Unassigned | Unassigned | Unassigned | Unassigned | 225420930 | 89.37 | Eastern Mediterranean sapropels |
| 16 | Archaeplastida | Streptophyta | Embryophyceae | Embryophyceae | Embryophyceae | Embryophyceae | 225420930 | 91.51 | Eastern Mediterranean sapropels |
| 17 | Opisthokonta | Fungi | Ascomycota | Saccharomycotina | Saccharomycetales | Metschnikowia | 225420832 | 100.00 | Eastern Mediterranean sapropels |
| 18 | Rhizaria | Cercozoa | Endomyxa-Ascetosporea | Paradinida | Paradinidae | Paradinium | 95115876 | 97.05 | Lost City hydrothermal field |
| 20 | Opisthokonta | Metazoa | Nematoda | Enoplea | Enoplea | Tripylina | 307647700 | 94.08 | Marine sediment |
| 21 | Rhizaria | Cercozoa | Endomyxa-Ascetosporea | Paradinida | Paradinida | Paradinida | 225420930 | 93.70 | Eastern Mediterranean sapropels |
| 22 | Rhizaria | Cercozoa | Endomyxa-Ascetosporea | Paradinida | Paradinida | Paradinida | 225420930 | 90.32 | Eastern Mediterranean sapropels |
| 23 | Opisthokonta | Metazoa | Arthropoda | Crustacea | Maxillopoda | Unassigned | 54401785 | 98.90 | - |
| 24 | Rhizaria | Cercozoa | Endomyxa-Ascetosporea | Paradinida | Paradinida | Paradinida | 194272484 | 96.00 | Rhizobium |
| 25 | Rhizaria | Cercozoa | Unassigned | Unassigned | Unassigned | Unassigned | 225420930 | 91.78 | Eastern Mediterranean sapropels |
| 27 | Alveolata | Dinophyta | Syndiniales | Dino-Group-II | Dino-Group-II-Clade-15 | Dino-Group-II-Clade-15 | 636661878 | 100.00 | East Pacific Rise 1500m |
| 28 | Rhizaria | Cercozoa | Endomyxa-Ascetosporea | Paradinida | Paradinidae | Unassigned | 194272483 | 97.99 | deep-sea colonization module Lucky Strike hydrothermal vent 1695 m |
| 29 | Opisthokonta | Metazoa | Nematoda | Chromadorea | Chromadorea | Theristus | 209553376 | 97.21 | - |
| 30 | Rhizaria | Cercozoa | Unassigned | Unassigned | Unassigned | Unassigned | 225420930 | 92.08 | Eastern Mediterranean sapropels |
| 31 | Unassigned | Unassigned | Unassigned | Unassigned | Unassigned | Unassigned | 225420930 | 91.23 | Eastern Mediterranean sapropels |
| 32 | Rhizaria | Cercozoa | Endomyxa-Ascetosporea | Paradinida | Paradinida | Paradinida | 225420930 | 89.86 | Eastern Mediterranean sapropels |
| 33 | Opisthokonta | Metazoa | Nematoda | Chromadorea | Chromadorea | Desmoscolex | 154795597 | 91.36 | - |
| 34 | Alveolata | Dinophyta | Dinophyceae | Dinophyceae | Dinophyceae | DinophyceaeX | 636663023 | 97.80 | Arctic Ocean 35m |
| 35 | Rhizaria | Cercozoa | Unassigned | Unassigned | Unassigned | Unassigned | 41584541 | 90.14 | - |
| 36 | Opisthokonta | Fungi | Unassigned | Unassigned | Unassigned | Unassigned | 225420930 | 92.33 | Eastern Mediterranean sapropels |
| 37 | Rhizaria | Cercozoa | Filosa-Thecofilosea | Cryomonadida | Cryothecomonas-lineage | Cryothecomonas | 225420930 | 90.96 | Eastern Mediterranean sapropels |
| 39 | Rhizaria | Cercozoa | Unassigned | Unassigned | Unassigned | Unassigned | 225420930 | 92.60 | Eastern Mediterranean sapropels |
| 40 | Rhizaria | Cercozoa | Endomyxa-Ascetosporea | Paradinida | Paradinida | Paradinida | 41584541 | 96.14 | Haplosporidian parasite of Pandalus platyceros |
| 41 | Opisthokonta | Metazoa | Nematoda | Enoplea | Enoplea | Halalaimus | 930579744 | 92.74 | Marine nematodes from Clarion-Clipperton Fracture |
| 43 | Rhizaria | Cercozoa | Endomyxa-Ascetosporea | Paradinida | Paradinida | Paradinida | 194272484 | 94.87 | Rhizobium |
| 44 | Hacrobia | Telonemia | Telonemia | Telonemia | Telonemia-Group-1 | Telonemia-Group-1 | 636631141 | 98.91 | coastal water around Qinhuangdao |
| 45 | Rhizaria | Cercozoa | Endomyxa-Ascetosporea | Paradinida | Paradinida | Paradinida | 225420930 | 91.37 | Eastern Mediterranean sapropels |
| 48 | Opisthokonta | Metazoa | Nemertea | Nemertea | Nemertea | Geonemertes | 225420930 | 93.48 | Eastern Mediterranean sapropels |
| 50 | Rhizaria | Cercozoa | Endomyxa-Ascetosporea | Paradinida | Paradinida | Paradinida | 225420930 | 89.65 | Eastern Mediterranean sapropels |
| 56 | Rhizaria | Cercozoa | Unassigned | Unassigned | Unassigned | Unassigned | 636659265 | 86.63 | Arctic Ocean, 500m |
| 57 | Alveolata | Ciliophora | Unassigned | Unassigned | Unassigned | Unassigned | 225420930 | 90.98 | Eastern Mediterranean sapropels |
| 59 | Rhizaria | Cercozoa | Filosa-Thecofilosea | Cryomonadida | Cryothecomonas-lineage | Cryothecomonas | 881206616 | 100 | Mudflat sediments in intertidal zone |
| 65 | Rhizaria | Cercozoa | Endomyxa-Ascetosporea | Paradinida | Paradinida | Paradinida | 225420930 | 95.34 | Eastern Mediterranean sapropels |
| 67 | Rhizaria | Cercozoa | Filosa-Thecofilosea | Cryomonadida | Protaspa-lineage | Protaspa-lineage | 529273906 | 98.92 | Surface seawater, estuarine continuum |
| 79 | Opisthokonta | Metazoa | Nematoda | Chromadorea | Chromadorea | Chromadorea | 854904390 | 93.41 | Slow sandfilter column for wastewater treatment, Leipzig |
| 80 | Rhizaria | Cercozoa | Unassigned | Unassigned | Unassigned | Unassigned | 225420930 | 91.01 | Eastern Mediterranean sapropels |
| 392 | Rhizaria | Cercozoa | Filosa-Thecofilosea | Cryomonadida | Cryothecomonas-lineage | Cryothecomonas | 225420930 | 93.15 | Eastern Mediterranean sapropels |
| 428 | Rhizaria | Cercozoa | Unassigned | Unassigned | Unassigned | Unassigned | 225420930 | 91.78 | Eastern Mediterranean sapropels |
| 561 | Opisthokonta | Metazoa | Platyhelminthes | Turbellaria | Acoelomorpha | Pseudaphanostoma | 378780946 | 90.62 | - |
| 666 | Rhizaria | Cercozoa | Filosa-Thecofilosea | Cryomonadida | Cryothecomonas-lineage | Cryothecomonas | 225420930 | 91.51 | Eastern Mediterranean sapropels |
| 761 | Rhizaria | Cercozoa | Endomyxa-Ascetosporea | Paradinida | Paradinidae | Paradinium | 95115876 | 98.52 | Hydrothermal field, Lost City |
| 878 | Rhizaria | Cercozoa | Unassigned | Unassigned | Unassigned | Unassigned | 225420930 | 92.6 | Eastern Mediterranean sapropels |
| 1193 | Rhizaria | Cercozoa | Filosa-Thecofilosea | Cryomonadida | Protaspa-lineage | Protaspa-lineage | 850483396 | 95.34 | Surface layer sediment from the East China Sea |
| 1216 | Rhizaria | Cercozoa | Endomyxa-Ascetosporea | Paradinida | Paradinidae | Paradinium | 95115876 | 92.88 | Lost City hydrothermal field |

References

1 Kuczynski, J. *et al.* Using QIIME to Analyze 16S rRNA Gene Sequences from Microbial Communities. *Current Protocols in Bioinformatics* **Unit 10.7**, doi:10.1002/0471250953.bi1007s36 (2011).

2 Cleary, D. F. R., Becking, L. E., Polónia, A. R. M., Freitas, R. M. & Gomes, N. C. M. Composition and predicted functional ecology of mussel-associated bacteria in Indonesian marine lakes. *A Van Leeuw J Microb* **107**, 821-834, doi:10.1007/s10482-014-0375-1 (2015).

3 Edgar, R. C. UPARSE: highly accurate OTU sequences from microbial amplicon reads. *Nature Methods* **10**, 996-998, doi:10.1038/nmeth.2604 (2013).

4 Wang, Q., Garrity, G. M., Tiedje, J. M. & Cole, J. R. Naïve Bayesian Classifier for Rapid Assignment of rRNA Sequences into the New Bacterial Taxonomy. *Appl. Environ. Microbiol.* **73**, 5261-5267, doi:10.1128/aem.00062-07 (2007).

5 Guillou, L. *et al.* The Protist Ribosomal Reference database (PR2): a catalog of unicellular eukaryote Small Sub-Unit rRNA sequences with curated taxonomy. *Nucleic Acids Res.* **41**, D597-D604, doi:10.1093/nar/gks1160 (2013).
